# Difference in the topography of atherosclerosis in the left versus right coronary artery in patients referred for coronary angiography

**DOI:** 10.1186/1471-2261-10-26

**Published:** 2010-06-10

**Authors:** George D Giannoglou, Antonios P Antoniadis, Yiannis S Chatzizisis, George E Louridas

**Affiliations:** 1 1st Cardiology Department, AHEPA University Hospital, Aristotle University Medical School, Thessaloniki, Greece

## Abstract

**Background:**

We sought to determine the difference in the localization of coronary artery disease (CAD) between the left and right coronary artery system and investigate the effect of sex and age on that difference.

**Methods:**

We retrospectively analyzed 17,323 consecutive angiographies from January 1^st^, 1984 to December 31^st^, 2003. The demographic parameters, in particular age and sex of the investigated cases as well as the angiographic results were recorded and summarized.

**Results:**

Of 13,305 cases with CAD, 861 (6.5%) had right coronary artery (RCA)-only disease, 4,621 (34.7%) had left coronary artery (LCA)-only disease, while 7,823 (58.8%) cases had concomitant RCA and LCA disease. LCA-only disease was more frequent than RCA-only disease [LCA-only/RCA-only odds ratio (OR): 5.37, 95% CI: 4.99 to 5.77, p < 0.001]. Women were more likely to have LCA-only disease (men/women OR 0.75 95% CI: 0.68 to 0.82, p < 0.001) compared with men who were more likely to present with concomitant RCA and LCA disease (men/women OR 1.33 95% CI: 1.21 to 1.45, p < 0.001). RCA-only and LCA-only disease were both more frequent in patients aged from 51 to 60 years, while concomitant RCA and LCA disease in patients between 61 and 70 years of age.

**Conclusions:**

LCA-only disease is more frequent than RCA-only disease. Men have a higher probability than women to present with concomitant RCA and LCA disease while women are more likely than men to be found with LCA-only disease.

## Background

The localization of coronary artery disease (CAD) in the right or left coronary arterial system exhibits diverse clinical presentation and prognosis. Acute occlusions due to lesions in the left coronary artery (LCA) manifest as left ventricular infarction while respective lesions in the right coronary artery (RCA) most commonly lead to isolated infarction of the right ventricle. The origin of culprit lesions for inferior and posterior wall infarctions depends on coronary dominance thus more often arising from the RCA [1,2]. The above clinical conditions show certain differences in their pharmacologic or interventional management, potential complications as well as patient follow-up. In this setting a further investigation of the frequency of CAD in the right or left coronary artery may be of interest.

In clinical practice, there is indirect evidence suggesting lower prevalence of atherosclerosis in the RCA than in the LCA, in particular with regards to unstable lesions. The main clinical manifestations of RCA occlusion, isolated right ventricular infarction and isolated left posterior wall infarction, are seen in only a minority of patients presenting with acute coronary syndrome [1,2]. A topographic predilection for CAD in the LCA especially in its proximal segments has been shown in several studies, the larger being a histopathologic study of nearly 3,000 cases [3]. Apart from this study, relevant data come either from autopsy [4,5] or limited scale clinical studies [6-8] and have not been validated in large populations. The aim of the present study was to determine the prevalence of CAD in the LCA as compared to the RCA in a large registry of patients referred for coronary angiography to our institution and investigate the effect of sex and age on that prevalence.

## Methods

Data of 17,323 consecutive patients who underwent coronary angiography for any reason in our institution from January 1^st^, 1984 to December 31^st^, 2003 were assessed through our database and retrospectively analyzed [9]. The demographic parameters, in particular age and sex of the investigated cases were recorded and summarized. All the angiograms were evaluated by an independent expert interventional cardiologist blinded to the patients' clinical and laboratory data.

We classified angiographic outcomes in 3 categories according to criteria used in previously published angiographic studies [10,11] i.e. (1) Angiographically significant stenoses, for one or more lesions causing a reduction of the coronary lumen diameter ≥50%; (2) Angiographically non-significant stenoses for coronary lumen obstructions <50%, and (3) Normal coronary arteries, referring to the absence of coronary obstruction in coronary angiography. We defined LCA-only disease as the presence of one or more angiographically significant stenoses in any of the following arteries: left main, left anterior descending, left circumflex or any of their branches, with normal RCA. Likewise, RCA-only disease was defined as one or more angiographically significant stenoses in the RCA or any of its branches, accompanied with normal LCA. To describe the age distribution of our subjects, they were divided in 7 age groups as shown in Table 1. The Institutional Medical Ethics Committee approved the study.

**Table 1:** Age distribution of the study subjects

*Age group (years)*	*Men (n)*	*Women (n)*	*Total (n)*
<30	97	14	111
31-40	559	61	620
41-50	2216	345	2561
51-60	4982	1287	6269
61-70	4345	1595	5940
71-80	1251	511	1762
>81	44	16	60

**Total**	**13494**	**3829**	**17323**

Categorical variables were summarized as absolute values and percentages. Comparisons between RCA and LCA disease were carried out using McNemar test, and were expressed as p values and odds ratios (OR) for discordant cells with the corresponding 95% confidence intervals (95% CI). Comparisons between men and women were performed using Pearson's chi-square test, and were expressed as p values and OR with the corresponding 95% CI. A value of p < 0.05 was considered statistically significant.

## Results

In our registry, 13,305 out of 17,323 cases (76.8%) were found with angiographically severe CAD. Of those cases, 861 (6.5%) had RCA-only disease, 4,621 (34.7%) had LCA-only disease, while the remaining 7,823 cases (58.8%) had concomitant RCA and LCA disease. Overall, LCA-only disease was more frequent than RCA-only disease (LCA-only/RCA-only OR: 5.37, 95% CI: 4.99 to 5.77, p < 0.001).

### Effects of Sex

The distribution of CAD in both sexes is shown in Table 2. Out of 11,156 men with significant stenoses, 720 (6.5%) were found with RCA-only disease, 3,752 (33.6%) with LCA-only disease and 6,684 (59.9%) with concomitant RCA and LCA disease. Out of 2,149 women with significant stenoses, 141 (6.6%) were found with RCA-only disease, 869 (40.4%) with LCA-only disease and 1,139 (53%) with concomitant RCA and LCA disease. LCA-only disease was more likely to occur in women than in men (men/women OR 0.75 95% CI: 0.68 to 0.82, p < 0.001). On the other hand, concomitant RCA and LCA disease was more likely to be found in men than in women, (men/women OR 1.33 95% CI: 1.21 to 1.45, p < 0.001). No difference was found in sex with regards to RCA-only disease.

**Table 2:** Prevalence of RCA and LCA disease in men and women

		*RCA-only*	*LCA-only*	*RCA & LCA*	*Total*
***Men (n)***		720	3752	6684	11156
***%***		6.5%	33.6%	59.9%	100.0%
***Women (n)***		141	869	1139	2149
***%***		6.6%	40.4%	53%	100.0%
***p***		0.79	<0.001	<0.001	
***OR***		0.98	0.75	1.33	
***95% CI***	***From***	0.82	0.68	1.21	
	***To***	1.18	0.82	1.45	

### Effects of Age

**Figure 1:**
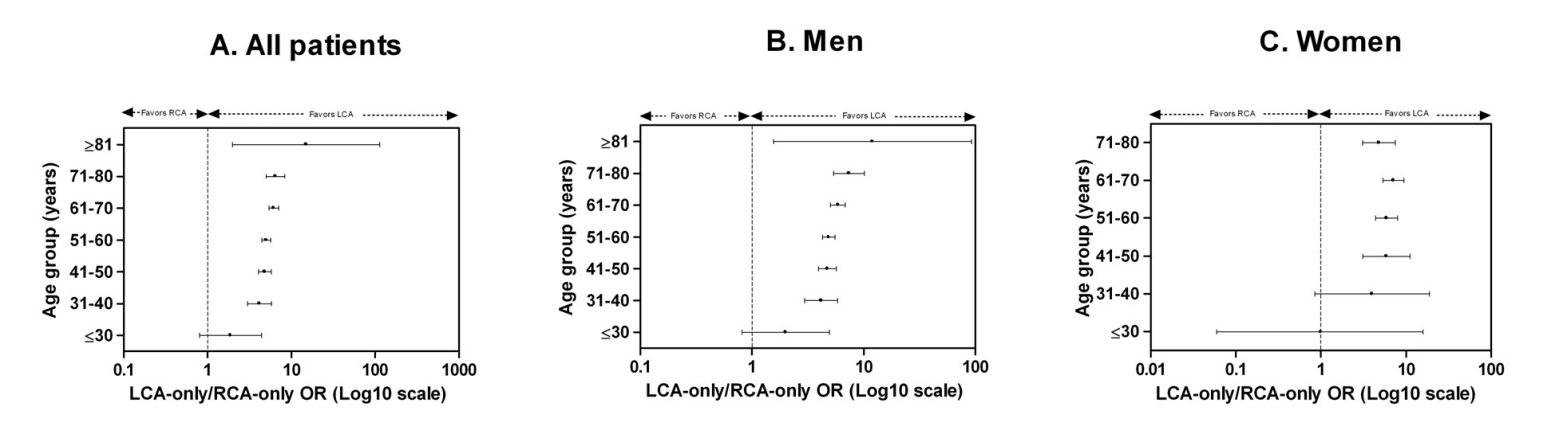
**LCA-only vs. RCA-only odds ratio (OR) plot for each decade of age**. Each point in the graph represents an OR, while the error bars correspond to its 95% confidence intervals (95% CI). An OR with 95% CI > 1.0 indicates significantly higher likelihood for LCA-only disease, whereas an OR with 95% CI < 1.0 indicates significantly higher likelihood for RCA-only disease. A. In all patients, LCA-only disease was more probable than RCA-only disease in all age groups except for subjects younger than 30 years of age where no significant difference was found. B. In men, LCA-only disease was more probable than RCA-only disease in all age groups except for subjects younger than 30 years of age where no significant difference was found. C. In women, LCA-only disease was more probable than RCA-only disease in all age groups except for subjects younger than 40 years of age where no significant difference was found. OR could not be calculated for subjects older than 80 years of age as no cases with RCA-only disease were noted in that age group.

LCA-only disease was more frequent than RCA-only disease in most age groups in both sexes (Figure 1A, B, C). RCA-only disease and LCA-only disease were most commonly found in patients from 51 to 60 years of age. On the other hand, cases with concomitant LCA and RCA disease most likely belonged to the older age group from 61 to 70 years (Table 3). For men, again RCA-only and LCA-only disease were most commonly found in patients from 51 to 60 years of age while concomitant LCA and RCA disease had similar frequency in patients from 51 to 60 and 61 to 70 years of age (Table 4). For women, all three categories of CAD localization were most frequent in the age group from 61 to 70 years (Table 5).

**Table 3:** Age distribution of RCA and LCA disease in patients with significant stenoses.

		Age group (years)						
		<30	31-40	41-50	51-60	61-70	71-80	>81
***RCA-only (n)***		8	44	153	340	247	68	1
***%***		0.9%	5.1%	17.8%	39.5%	28.7%	7.9%	0.1%
***LCA-only (n)***		15	183	738	1707	1522	441	15
***%***		0.3%	4.0%	16.0%	36.9%	32.9%	9.5%	0.3%
***RCA & LCA (n)***		5	167	995	2695	2960	969	32
***%***		0.1%	2.1%	12.7%	34.4%	37.8%	12.4%	0.4%
***LCA-only/RCA-only OR***		1.88	4.16	4.82	5.02	6.16	6.49	15
***95% CI***	***From***	0.8	2.99	4.05	4.47	5.39	5.02	1.98
	***To***	4.42	5.78	5.74	5.64	7.05	8.37	113.56

***p***		0.21	<0.001	<0.001	<0.001	<0.001	<0.001	<0.001

**Table 4:** Age distribution of RCA and LCA disease in men with significant stenoses.

		Age group (years)						
		<30	31-40	41-50	51-60	61-70	71-80	>81
***RCA-only (n)***		7	42	142	291	193	44	1
***%***		1.0%	5.8%	19.7%	40.4%	26.8%	6.1%	0.1%
***LCA-only (n)***		14	175	673	1416	1137	325	12
***%***		0.4%	4.7%	17.9%	37.7%	30.3%	8.7%	0.3%
***RCA & LCA (n)***		5	161	938	2410	2403	739	28
***%***		0.1%	2.4%	14.0%	36.1%	36.0%	11.1%	0.4%
***LCA-only/RCA-only OR***		2	4.17	4.74	4.87	5.89	7.39	12
***95% CI***	***From***	0.81	2.98	3.95	4.29	5.06	5.39	1.56
	***To***	4.96	5.84	5.68	5.52	6.86	10.12	92.29

***p***		0.19	<0.001	<0.001	<0.001	<0.001	<0.001	<0.01

**Table 5:** Age distribution of RCA and LCA disease in women with significant stenoses.

		Age group (years)						
		<30	31-40	41-50	51-60	61-70	71-80	>81
***RCA-only (n)***		1	2	11	49	54	24	0
***%***		0.7%	1.4%	7.8%	34.8%	38.3%	17.0%	0.0%
***LCA-only (n)***		1	8	65	291	385	116	3
***%***		0.1%	0.9%	7.5%	33.5%	44.3%	13.3%	0.3%
***RCA & LCA (n)***		0	6	57	285	557	230	4
***%***		0.0%	0.5%	5.0%	25.0%	48.9%	20.2%	0.4%
***LCA-only/RCA-only OR***		1	4	5.91	5.94	7.13	4.83	n/a
***95% CI***	***From***	0.06	0.85	3.12	4.39	5.36	3.11	n/a
	***To***	15.99	18.84	11.2	8.04	9.48	7.50	n/a

***p***		1.5	0.10	<0.001	<0.001	<0.001	<0.001	0.25

## Discussion

We conducted an observational, retrospective, single-center study to evaluate the differences in the topography of CAD in the right as opposed to the left coronary arterial system in a large dataset of 17,323 patients who underwent coronary angiography during a 20-year period. The present study is the first large scale registry to demonstrate that LCA-only disease was more frequent than RCA-only disease. Our results of higher angiographic localization of atherosclerosis in LCA as compared to RCA further confirm previous findings derived from histopathologic (n = 2,964) [3], angiographic (n = 302) [6], intravascular ultrasound (n = 262) [8] and computed tomography (n = 102) [7] data. Furthermore, our results are in agreement with clinical evidence that isolated right ventricular or left posterior infarction, which are basically attributed to RCA lesions, are less common than left ventricular infarctions [1,2].

Local hemodynamic and anatomic particulatiries of LCA vs RCA may be responsible for the predilection of atherosclerosis development in the left coronary system [12,13]. First, the right coronary flow is more uniform during the cardiac cycle as compared to the left, which experiences a remarkable systolic decline accompanied by a significant diastolic increment [12,14,15]. As a result the local endothelial shear stress (ESS) in LCA is lower and more oscillatory, especially in atherosclerosis-prone regions, as compared to RCA [12]. Low and oscillatory ESS shift the endothelial cell function and structure to a pro-atherosclerotic phenotype promoting atherosclerosis [12,13,16]. Second, increased wall stress is another potent atherogenic stimulus [17]. LCA segments are exposed to higher wall stress during systole than RCA. This is a result of the different contractile properties of the left versus right ventricle [12,17]. The greater wall stress throughout the cardiac cycle in LCA may form an atherogenic stimulus [12]. Third, RCA and LCA have differences in anatomy. Left anterior descending artery exhibits twice the torsion of RCA [18]. Torsion may play a major role in generating helical flow patterns, which may promote atherosclerosis progression [13,19,20]. Finally, the increased branching of the LCA as compared to the RCA contributes to the development of disturbed flow in the respective regions thus rendering a more atherosclerosis-susceptible environment in the LCA [21].

Our finding of a higher probability of men to present with concomitant RCA and LCA disease may indicate that male sex is prone to a more widespread form of atherosclerosis [22]. On the other hand, the increased likelihood of women to present with LCA-only disease may in part explain their worse prognosis after a coronary event [23].

Concerning the effects of age in the localization of CAD, our findings suggest that concomitant RCA and LCA disease is found at older ages than isolated RCA or LCA involvement. This comes into consistence with previous autopsy studies exhibiting an increased prevalence of CAD with the progression of age [4,5]. In women however, all three categories of CAD were most prevalent in an older age group than in men, and this is attributed to the established tendency for women to develop CAD at a later age than men [24].

### Study Limitations

Several limitations apply within our study. First, it was performed to a specific group of patients referred for coronary angiography; thus, the generalizability of our results to the general population is limited and the real prevalence of the localization of CAD in the community remains unknown. Also, a referral bias regarding the clinical presentation of the patients cannot be excluded as the study was conducted in a tertiary care center. Detailed information on the medical history of the subjects was not available due to heterogeneity of cases studied in terms of the cardiology center they were referred from and due to the lack of electronic patient records for the first years of the study. These, in association with the large number of study subjects and the long period in the past our report extends to, render not feasible to investigate on potential differences with regards to coronary artery disease risk factors. Also, we were unable to assess variations in the localization of atherosclerosis within the proximal or distal parts of the coronary arteries, although there is evidence that most thin-cap fibroatheromas and ruptured plaques are found in the proximal third of the coronaries [25]. The burden of CAD in human coronaries was based on data from conventional angiography, although newer imaging modalities, such as intravascular ultrasound, have been proved more accurate in imaging of the anatomy and extent of CAD [26]. Finally, the unavailability of follow up data did not permit us to evaluate the long-term outcome of CAD in relation to its localization.

## Conclusions

In conclusion, this study attempted to provide an anatomic description of the localization of CAD in a large population referred for cardiac catheterization and to examine possible differences in atherosclerosis between RCA and LCA. LCA-only disease was more common than RCA-only disease across all ages. LCA-only disease was more likely to occur in women, while concomitant RCA and LCA disease to men. Further studies are needed to investigate the factors which may account for the variation in the localization of coronary atherosclerosis.

## Competing interests

The authors declare that they have no competing interests.

## Authors' contributions

GG conceived of the study and contributed to the design of the study protocol, the coordination of data collection and interpretation and the revision of the manuscript. AA contributed to the design of the study protocol, performed the statistical analyses of data and drafted the manuscript. YC contributed to the design of the study protocol and revised the final manuscript, particularly in regard to review of the literature. Finally, GL participated in the study design and study coordination as well as the final evaluation of the submitted manuscript. All authors read and approved the final manuscript.

## Pre-publication history

The pre-publication history for this paper can be accessed here:

http://www.biomedcentral.com/1471-2261/10/26/prepub

## References

[B1] Andersen HR Falk E Nielsen D Right ventricular infarction: frequency, size and topography in coronary heart disease: a prospective study comprising 107 consecutive autopsies from a coronary care unit J Am Coll Cardiol 1987 10 1223 1232 10.1016/S0735-1097(87)80122-5 3680789

[B2] Brady WJ Erling B Pollack M Chan TC Electrocardiographic manifestations: acute posterior wall myocardial infarction J Emerg Med 2001 20 391 401 10.1016/S0736-4679(01)00318-3 11348821

[B3] Montenegro MR Eggen DA Topography of atherosclerosis in the coronary arteries Lab Invest 1968 18 586 593 5681200

[B4] Ackerman RF Dry TJ Edwards JE Relationship of Various Factors to the Degree of Coronary Atherosclerosis in Women Circulation 1950 1 1345 1354 15414552 10.1161/01.cir.1.6.1345

[B5] White NK Edwards JE Dry TJ The Relationship of the Degree of Coronary Atherosclerosis with Age, in Men Circulation 1950 1 645 654

[B6] Halon DA Sapoznikov D Lewis BS Gotsman MS Localization of lesions in the coronary circulation Am J Cardiol 1983 52 921 926 10.1016/0002-9149(83)90506-4 6637847

[B7] Schmermund A Baumgart D Mohlenkamp S Kriener P Pump H Gronemeyer D Seibel R Erbel R Natural history and topographic pattern of progression of coronary calcification in symptomatic patients: An electron-beam CT study Arterioscler Thromb Vasc Biol 2001 21 421 426 11231923 10.1161/01.atv.21.3.421

[B8] Tuzcu EM Kapadia SR Tutar E Ziada KM Hobbs RE McCarthy PM Young JB Nissen SE High prevalence of coronary atherosclerosis in asymptomatic teenagers and young adults: evidence from intravascular ultrasound Circulation 2001 103 2705 2710 11390341 10.1161/01.cir.103.22.2705

[B9] Giannoglou GD Antoniadis AP Chatzizisis YS Damvopoulou E Parcharidis GE Louridas GE Prevalence of narrowing >or = 50% of the left main coronary artery among 17,300 patients having coronary angiography Am J Cardiol 2006 98 1202 1205 10.1016/j.amjcard.2006.05.052 17056328

[B10] Marroquin OC Kip KE Kelley DE Johnson BD Shaw LJ Bairey Merz CN Sharaf BL Pepine CJ Sopko G Reis SE Metabolic syndrome modifies the cardiovascular risk associated with angiographic coronary artery disease in women: a report from the Women's Ischemia Syndrome Evaluation Circulation 2004 109 714 721 10.1161/01.CIR.0000115517.26897.A7 14970105

[B11] Budoff MJ Yang TP Shavelle RM Lamont DH Brundage BH Ethnic differences in coronary atherosclerosis J Am Coll Cardiol 2002 39 408 412 10.1016/S0735-1097(01)01748-X 11823077

[B12] Chatzizisis YS Giannoglou GD Parcharidis GE Louridas GE Is left coronary system more susceptible to atherosclerosis than right? A pathophysiological insight Int J Cardiol 2007 116 7 13 10.1016/j.ijcard.2006.03.029 16908081

[B13] Chatzizisis YS Coskun AU Jonas M Edelman ER Feldman CL Stone PH Role of endothelial shear stress in the natural history of coronary atherosclerosis and vascular remodeling: molecular, cellular, and vascular behavior J Am Coll Cardiol 2007 49 2379 2393 10.1016/j.jacc.2007.02.059 17599600

[B14] Chatzizisis YS Giannoglou GD Pulsatile flow: a critical modulator of the natural history of atherosclerosis Med Hypotheses 2006 67 338 340 10.1016/j.mehy.2006.02.005 16546326

[B15] Chatzizisis YS Giannoglou GD Matakos A Basdekidou C Sianos G Panagiotou A Dimakis C Parcharidis GE Louridas GE In-vivo accuracy of geometrically correct three-dimensional reconstruction of human coronary arteries: is it influenced by certain parameters? Coron Artery Dis 2006 17 545 551 10.1097/00019501-200609000-00008 16905967

[B16] Chatzizisis YS Jonas M Coskun AU Beigel R Stone BV Maynard C Gerrity RG Daley W Rogers C Edelman ER Prediction of the localization of high-risk coronary atherosclerotic plaques on the basis of low endothelial shear stress: an intravascular ultrasound and histopathology natural history study Circulation 2008 117 993 1002 10.1161/CIRCULATIONAHA.107.695254 18250270

[B17] Thubrikar MJ Robicsek F Pressure-induced arterial wall stress and atherosclerosis Ann Thorac Surg 1995 59 1594 1603 10.1016/0003-4975(94)01037-D 7771858

[B18] Ding Z Zhu H Friedman MH Coronary artery dynamics in vivo Ann Biomed Eng 2002 30 419 429 10.1114/1.1467925 12085995

[B19] Zhu H Ding Z Piana RN Gehrig TR Friedman MH Cataloguing the geometry of the human coronary arteries: A potential tool for predicting risk of coronary artery disease Int J Cardiol 2008 18597872 10.1016/j.ijcard.2008.03.087 PMC2759354

[B20] Van Langenhove G Wentzel JJ Krams R Slager CJ Hamburger JN Serruys PW Helical velocity patterns in a human coronary artery: a three-dimensional computational fluid dynamic reconstruction showing the relation with local wall thickness Circulation 2000 102 E22 24 10899104 10.1161/01.cir.102.3.e22

[B21] Feldman CL Stone PH Intravascular hemodynamic factors responsible for progression of coronary atherosclerosis and development of vulnerable plaque Curr Opin Cardiol 2000 15 430 440 10.1097/00001573-200011000-00010 11198626

[B22] Lerner DJ Kannel WB Patterns of coronary heart disease morbidity and mortality in the sexes: a 26-year follow-up of the Framingham population Am Heart J 1986 111 383 390 10.1016/0002-8703(86)90155-9 3946178

[B23] Schwartz LM Fisher ES Tosteson NA Woloshin S Chang CH Virnig BA Plohman J Wright B Treatment and health outcomes of women and men in a cohort with coronary artery disease Arch Intern Med 1997 157 1545 1551 10.1001/archinte.157.14.1545 9236556

[B24] Roeters van Lennep JE Zwinderman AH Roeters van Lennep HW Westerveld HE Plokker HW Voors AA Bruschke AV van der Wall EE Gender differences in diagnosis and treatment of coronary artery disease from 1981 to 1997. No evidence for the Yentl syndrome Eur Heart J 2000 21 911 918 10.1053/euhj.1999.1941 10806015

[B25] Cheruvu PK Finn AV Gardner C Caplan J Goldstein J Stone GW Virmani R Muller JE Frequency and distribution of thin-cap fibroatheroma and ruptured plaques in human coronary arteries: a pathologic study J Am Coll Cardiol 2007 50 940 949 10.1016/j.jacc.2007.04.086 17765120

[B26] Schoenhagen P Nissen S Understanding coronary artery disease: tomographic imaging with intravascular ultrasound Heart 2002 88 91 96 10.1136/heart.88.1.91 12067962 PMC1767193

